# Frames and comparators: How might a debate on synthetic biology evolve?

**DOI:** 10.1016/j.futures.2013.02.002

**Published:** 2013-04

**Authors:** Helge Torgersen, Markus Schmidt

**Affiliations:** a Institute of Technology Assessment, Austrian Academy of Sciences, Strohgasse 45, 1030 Vienna, Austria; b Biofaction KG, Grundsteingasse 36/41, 1160 Vienna, Austria

## Abstract

A stimulated early public debate is frequently advocated when introducing an emerging technology like synthetic biology (SB). To debate a still quite abstract technology, participants functionally need a frame that determines which arguments are legitimate and which issues are relevant. Often, such frames are based on previous debates over other novel technologies. Three technologies currently provide frames for discussing SB: (green) biotechnology, nanotechnology and information technology. In the biotechnology debate, risk has long been emphasised over economic benefits. More recently, nanotechnology has been referred to mostly in terms of benefits, while risks tended to be an issue for scientific discourses. This has frequently been related to the many outreach activities around nanotechnology. Information technology, finally, has retained the image of being ‘cool’ and useful on a personal level. The technology itself is taken for granted and only the consequences of particular applications have been up for discussion. Upstream engagement exercises in SB will have to consider the comparator chosen more diligently, because it might influence the debate on SB ‘out there’ in the long run.

## Highlights

► Upstream public engagement in synthetic biology is advocated with different rationales in mind. ► A debate on novel technology needs a comparator to provide a perspective. ► Subject to the comparator, different frames govern a debate and influence the outcome. ► For synthetic biology, comparators are bio-, nano- and information technology. ► Choosing the comparator not only implies procedural but normative choices.

## 1 Synthetic biology and public engagement

Synthetic biology (in the following SB), one of the most prominent emerging technologies is a vibrant field of research and development that is still little known among the public [1]. Like with other emerging technologies, proponents keep promising steep progress and ensuing technological and economic benefits, while a number of security, safety and ethical aspects are considered contentious [2].

In the past, technologies such as nuclear power [3] or agricultural biotechnology [4] elicited public controversies mostly over the issue of risk. Other emerging technologies such as medical procedures based on stem cells met with moral objections against this form of research [5,6]. Many other technologies got implemented mostly under the perspective of economic gains that they would bring. However, economic issues had been major reasons of conflict in past technology debates already under the industrial revolution. Conflict over technologies and how to deal with it has been a recurrent issue for a long time [7].

Pubic debate has been advocated as a means to rationally deal with technology conflicts. Investigating opportunities to open up a public debate, Stirling [8] referred to three approaches why experts think such a debate might help in mitigating a conflict: an instrumental, a normative and an substantive one [9].

The instrumental approach might be illustrated by a frequently encountered practice from within the research community. Drawing the analogy to past conflicts, members of this community fear that aspects of the technology considered contentious could give rise to controversies and thus, jeopardise its prospects. The controversy over GM food mostly serves as a blueprint for the fear of public unease, and the lack of an adequate, fact-based public debate is identified as one of the main reasons for public rejection.

This is a pattern also encountered in the case of SB. For example, the European Scientific Academies Advisory Council recommended public dialogue to prevent a potentially pending public rejection [10]. Only if introduced to the technology and its scientific underpinnings early on, so the argument, the public would be able to reject false risk claims. In their statement, EASAC “favour(s) the establishment of a dialogue between scientists and the public on the future of the technology and its potential benefits. Such an exchange of views, based on evidence, offers the best hope of creating a context in which the public can realistically assess the fears expressed in more sensationalist accounts.” [10, p. 2]. The argument largely followed the popular but somewhat out-dated ‘public understanding of science’ rationale (critically [11]), which claims that fears of technology risks largely result from a lack of individual factual knowledge. The rationale is outlined in the full report: “In engaging with, and encouraging, public debate about the opportunities and challenges, the academies […] have a particular responsibility. This is to ensure that regulations are not introduced that will – […] – stifle research.” [12, p. 2]. To save the research community from obstacles resulting from a possibly negative public opinion was a main concern, and early dialogue was seen as an appropriate means to this end.

The normative approach can be illustrated by the quest for public deliberation from the US Presidential Commission for the Study of Bioethical Issues [13, p. 152], who stated that “Public deliberation is particularly valuable while the field is still young, as there is a unique opportunity to shape its development in ways most likely to promote the public good while assuring safety and security.” Regarding the underlying rationale, the PCSBI emphasised fundamental principles: “The principle of democratic deliberation reflects an approach to collaborative decision making that embraces respectful debate of opposing views and active participation by citizens.” And further: “The principle of democratic deliberation highlights the importance of robust public participation in both the development and implementation of specific policies as well as in a broader, on-going national conversation about science, technology, society, and values.” (p. 151) Thus, the argumentation highlights early collaborative decision making as a principle and end in itself.

The substantive approach encapsulates much of what advocates of the social shaping of technology find attractive with public debate. They argue that if the implications of the new technology would become visible only upon its wide distribution, for example in the form of product hazards, it would be too late to influence the then firmly established technology trajectory (for past hazards see [14]; for SB: [15]). Situated and lay knowledge has to complement abstract expert knowledge that might be blind to problems caused by the technology in an everyday setting [16]. Therefore, lay or stakeholder participation as well as an open debate over potential implications at an early stage should influence the technology in a socially beneficial way when this is still possible (for nanotechnology [17]). This argument aims at gains in rationality, shaping technology along the interests of society at large (however determined).

In times of ‘technoscience’, however, where basic research and technology development merge [18], this can only be done at an early stage of development, where applications have not yet reached the market. In such a view, the more classical argument that the market should decide over consumer preferences only after products were available is misplaced. Rather, the possible trajectories of the technology and their alternatives should be made visible.

Taken together, a common interest although from different reasons seems to exist among various stakeholders and scholars to somehow involve the public early in technological innovation. In other words, they agree that the public needs to be engaged ‘upstream’ [19], without referring to how this should be done. The task is challenging as “Innovation-related dialogue or engagement is particularly difficult […] when there is ignorance or uncertainty *among all parties involved* about the eventual nature of new products, processes, benefits and risks.”^1^ [20, p. 38, original emphasis]. It is uncertainty that makes discussions difficult – what is it that needs to be debated, and why? A public debate of some sort usually does not exist because with emerging technologies, arguments have to be developed over incalculable consequences from procedures and products that do not yet exist or never may come into life.

In its absence, exercises in upstream public engagement have been proposed and held, with a view to indicate the subject and thrust of a broader public debate to be stimulated. However, the discussions in a deliberately set-up engagement exercise cannot be considered necessarily similar to unplanned debates ‘out there’ in the public sphere.^2^ The latter arise bottom-up and tend to involve all sorts of experts, stakeholders and members of the public in an entirely uncoordinated way. In contrast, upstream public engagement exercises are deliberately organised, often by STS social scientists or specialists in technology assessment in the form of distinct participatory projects [22].

Relying on external funding, the organisers have to argue why they are held on what issue and under which conditions. Apart from more official justifications, a number of tricky, seemingly organisational questions arise; for example, in which form should the engagement take place, and what kind of debate or dialogue^3^ should be stimulated? Who should take part in it, how should the relevant issues and the legitimate arguments be determined, and what could be appropriate comparisons? Answers to these questions are not only instrumental to conduct a debate; rather, they incorporate far-reaching normative choices. They not only will influence the course and outcome of the engagement exercise but might also propose, if successful, what issues and arguments might become relevant in an ensuing broader public debate.

In the following, we will argue that – influenced by upstream engagement exercises – a public debate over SB ‘out there’ might develop very differently subject to issues and arguments determined to be relevant and the choice what SB might be compared to early-on. This choice might heavily influence the future public image of the technology and impinge on its commercial success.

We do not, however, aim at proposing better ways of holding a debate, more effectively influencing technology development upstream or devising better governance measures. Neither do we argue for or against public participation in the shaping of technology nor support a particular view what interest(s) research, technology development and implementation should serve in the first place. We only observe that these days, obviously very different interests seem to merge in a sometimes not fully reflected quest for upstream engagement and public debate. Hence, stimulating and conducting such a debate requires normative choices even where they seem to be technical only.

## 2 The role of frames and comparators

It has long been known that every debate is subject to a dominant frame; otherwise it would be impossible to discuss anything. In other words, without a shared frame there is indifference rather than disagreement [23]. The underlying logic is that in order to debate anything it is necessary to develop a common understanding of what is considered relevant and which form of argumentation is deemed legitimate before arguing over factual as well as normative positions. If such an understanding cannot be developed a debate cannot be held – potential participants would be unable to agree over what to discuss. They have to find a common frame to find a basis on which conflicts can be conducted [24].

The term ‘frame’ has been used in different ways; often, it refers to diverging and often normatively tainted arguments that emerge under a debate (see e.g. [25]). Focussing on the *content* of arguments results in a frame concept as a classification of different normative positions. In contrast, analysing the implicit discursive *rules* results in a frame concept that emphasises patterns guiding participants how to perform a debate. This is a perspective informed by the work of Erving Goffman [26] who tried to uncover the implicit but commonly shared patterns of structuring discourses. Following this line of thought, we consider frames to be powerful organisation principles of individual perceptions and interpretations providing a common discursive basis for a debate. The function of a frame is thus to create a shared discursive basis on which conflicts can be conducted. They determine the fundamental rules of the discourse rather than whether something is considered good or bad.

From this perspective, the implicit power effects of a frame become visible. On the one hand, frames direct and structure habits of seeing, thinking, and acting; on the other hand, they determine controversies to the extent that adversaries must refer to an established frame in a constructive way. If a frame becomes dominant, powerful rules for the organisation of conflict communication are established and particular issues and arguments become relevant. Issues of concern must fit the dominant frame; if not, they will be neglected. However, issues suppressed can reappear in different guises after having been adjusted to the dominant frame, or survive without being really processed. For example, the cultivation of genetically modified crops has mainly been discussed under a risk frame, despite many economic implications [27], which contributed to the controversy persisting.

The choice of a dominant frame does not pre-empt the outcome of a debate. However, it has implications for the choice of the relevant expertise, the range of stakeholders to be included, the kind of measures to be taken and many other governance aspects. As mentioned above, the debate on green biotechnology was mostly held under a risk frame, i.e. arguments about risk for human health and the environment were deemed more relevant than those related to economic equality or ethical concerns. Consequently, scientists were asked to provide risk probabilities and prior assessments were made mandatory (in Europe) to identify risks. In the stem cell debate, an ethics frame prevailed, and arguments over the sanctity of embryonic life were considered more salient than potential health risks. The expertise taken on board included those of ethicists and clergymen, and measures included a ban on some forms of research [24].

Apart from risk and ethics frames we also find an economic frame, emphasising the opportunities for future benefits or, alternatively, the possible losses in case of non-implementation. It may also cover alternative ways at arriving at a similar (economic) outcome. This frame is adopted almost by default in most questions of technology implementation as it falls easy to bring in line with conventional cost-benefit analyses and other economic tools usually prevalent in technology policy. While an economic frame almost by default prevails in the discussion of non-controversial technologies, this is not necessarily the case when it comes to debates over the implementation of a controversial technology. However, under an economic (benefit) frame technology implementation is not supported at any rate; rather, this frame may also govern opposing arguments pertaining non-equitable benefit distribution, for example.

In principle, other frames than risk, ethics and the economy might be conceivable. However empirically, in technology debates they are most frequent: media analyses of technology controversies [28] revealed ‘basic frames’ covering, in slightly other words, the three frames addressed above but no fundamentally different ones.^4^

For any upstream debate on an emerging technology, the dominant frame does not emerge automatically as the issue is still vague in its properties and consequences. Past experiences with then new technologies, such as agricultural biotechnology, had shown that setting a frame for the debate often implied establishing power relations.^5^ However today, exercising power openly does not seem to be considered adequate any more, not the least under the catchword of ‘responsible innovation’ [30]. The debate is open in principle; in fact, initially it serves to explore a dominant frame all participants would agree to. This openness might offer advantages, but it implies looking at experimental work performed today to determine speculative future implications [17]. This not only is challenging for a lay audience but also for the scientists involved, who rarely can foresee the implications of their developments in society.

Analogies to other technologies having left a mark in the publics’ imagination come in handy here. It is to no wonder that debates over possible futures involving emerging technologies are mostly grounded in debates over present day's controversial technologies [31]; thus, the new technology is implicitly (or explicitly) compared to an older one. In fact, it is almost inevitable to refer to such a comparator in order to imagine possible consequences desired or to be avoided. A comparator provides guidance in getting to terms with the promises and threats of a new technology. It bundles different expectations into a common guiding image [32] at the intersection of hopes and fears. As a point of departure, expectations guide the debate in a particular direction and provide commonly shared aspects and perspectives [33].

In doing so, the frames dominant in the past debate on the older comparator technology influence the frames developing in the debate over the new technology.^6^ In practice, frames are often copied from the comparator debate and pasted into the new one: dominant arguments and the choice of issues serve as a blueprint or a point of departure for debating the implications of the new technology.

The choice of a comparator may also imply a reification of the new technology, determining how the debate is going to be held. Wynne [34] argued that an early influence may in fact ‘stage’ a debate. Adopting a comparator may be considered such an influence. Which technology an emerging one should be compared to is not only a question of the respective ‘nature’ and hence, of the assumed similarities. Rather, it is highly relevant which discipline and scientific or engineering community is dominant and what important members think of the technology. In other words, the dominant culture of the scientific community developing the technology shapes the frame a technology is being debated under – even if the developers’ efforts are directed at warding off potential critique pro-actively.^7^

With culture we not only mean the way scientists and engineers see their role in the development process. Equally important is how they think their science and engineering activities and the ensuing products relate to society as a whole. This includes how they think society will relate to the products, the processes giving rise to them and the people standing behind.

The culture of a scientific and engineering community also influences how the similarities between two technologies are defined. This again plays a role in determining which comparator technology would be considered adequate. Hence, there is a chicken-and-egg relation between comparators and frames: the comparator adopted indicates that a particular frame is preferred. At the same time, the dominant frame effectively influences the choice of a comparator. All this has to be seen in a dynamic perspective: in most cases, frames and comparators evolve under the first rounds of a debate. Often, they appear, or are depicted, as a matter of fact, as if no other choice would be possible. But even if voluntarily set in the beginning, they have to prove their usefulness and may be dismissed or supplemented later if the debate makes a turn. This particularly applies to debates ‘out there’ in the public sphere, where any attempt at controlling them has proven futile.

Synthetic biology has been linked (or traced back [15]) to different comparator technologies: biotechnology, nanotechnology and information technology [35]. The respective links are being forged more explicitly in various non-scientific reports by NGOs (e.g. [36]) or academics (e.g. [10,37]) and more implicitly in the media (e.g. [38,39]. Each comparator conveys different aspects, expectations, hopes and fears, and the dominant debates are held under partly or entirely different frames, respectively. The analogies drawn give a paradigmatic picture of divergent or sometimes even incompatible technology trajectories. As yet it is uncertain which of them (or maybe another one) would turn out to be dominant in a broader public debate to come.

## 3 Comparing synthetic biology

### 3.1 Biotechnology

Genetically engineered (GM) plants and food, i.e. agricultural or ‘green’ biotechnology, have been subject to adverse public perception in some countries. It is therefore not astonishing that critical NGOs referred to genetic engineering as a comparator for SB [36], painting a dark picture with SB being internalised into the future agenda of big business to exploit natural resources even more aggressively. The ETC Group dubbed SB to be ‘extreme genetic engineering’ and invented the nickname ‘Synthia’ for Craig Venter's alleged bacterial creation. However, few other major NGOs have taken notice of SB so far. Obviously they do not consider the issue important or suitable enough for campaigning. Another explanation is that anti-biotechnology campaigns already had exploited the arsenal of negative scenarios so that there is little left to campaign on SB [40]. As a general rule, those environmental NGOs having addressed SB so far tended to extrapolate arguments against various forms of biotechnology to future applications of SB.

Analyses of German and Austrian media showed that SB was addressed as a new form of biotechnology [39]. Rather than the technology as such, individual protagonists such as Craig Venter were prominent subjects of media reports and ambivalently described as biotechnologists. Risks and moral problems known from the biotechnology debate were taken up again, but expectations for potential benefits prevailed. Metaphors centred on engineering and playing (e.g. ‘Lego bricks’). Interestingly, allusions to ‘playing God’ were less frequent than bioethics experts would have claimed [41,42].

Policy refers to the (green) biotechnology comparator mostly in the form of a menace: ‘the same’ as with GM food (i.e. a failed implementation due to public rejection) must be prevented. The IRGC (37, p. 38) described this reaction common among experts and policy makers as “… the ‘fear of the fear of the public’ – a concern among those working on synthetic biology that the kind of public response to GM crops that emerged in Europe in the late 1990s would be transferred, perhaps in a more virulent form, to synthetic biology. “The problem, according to the IRGC, lies in how to “… find ways of reconciling fundamentally conflicting values or ideologies”. The report anticipates that “…there are strong differences of opinion at the outset of a debate, it is hard to manage the process in such a way as to avoid further polarisation of views and exacerbation of conflict.” (37, p. 37) A regulatory expert's view on the GMO controversy may be seen lurking through the lines.

Ethical considerations are important issues expected to elicit negative public reactions (see [2]). Transgressing the boundary between artefacts and living organisms and the prospects of ‘creating life’ are often considered prone to conflict. In addition, experts from various disciplines emphasise biosecurity (prevention of intended harm) and biosafety (prevention of unintended harm). As in other areas of biotechnology, European and US perceptions seem to differ here: US accounts addressed risk mostly as being confined to biosecurity, i.e. bioterror attacks. European reports tend to emphasise biosafety as well, not unlike agricultural biotechnology [43].

Communication strategies by scientists and industry on the one hand try to emphasise the difference to conventional biotechnology aka genetic engineering. This may be related to presenting a promising new field to funding agencies. On the other hand, many scientists consider SB an extension to genetic engineering proceeding towards artificialness [44]. In their approach to the public, allusions to the biotechnology conflict in Europe can be found although many prominent scientists come from the US where biotechnology has not met with particular problems among the public. In Europe, SB has not met with strong objections so far despite fears among policy makers. A reason might be that so far, it has not impinged on food, which used to be a major conflict trigger with various technologies.^8^

### 3.2 Nanotechnology

Like biotechnology, nanotechnology is both a comparator and an element that co-constitutes SB. It is one of the technologies that are said to ‘converge’ with biotechnology and cognitive sciences to form something new. Dealing with elements in the range of biologically active structures, there is an overlapping field in nanobiotechnology, although the communities are rather separate [35].

Nanotechnology is an emerging technology *par excellence*, met with great expectations and benefiting from massive public funding – the European Commission alone spent €3.5 billions through FP7. Potential risk concerns have been addressed more professionally than with biotechnology in its early days, i.e. risk concerns were taken more seriously and triggered investigations instead of being dismissed as ‘unscientific’. Assessments mostly resulted in identifying far-reaching knowledge gaps to be filled-in incrementally but rapidly.^9^ In contrast to the perception of some technical experts and policy makers, press coverage has not particularly focussed on risk so far; rather, the potentials for huge benefits have been mostly to the fore [45]. Apart from occasional demonstrations mostly in France, technology critics did not succeed in mobilising significant parts of the public despite many speculations that nanotechnology might elicit concerns similar to green biotechnology.^10^

Such speculations may have contributed to setting up a variety of public engagement exercises. Apart from information initiatives such as the ‘Nanotruck’ in Germany, science fairs and similar outreach activities, a number of participatory events of different guises were seen as constituting a new way of introducing a technology. In the events, concerns and problems were addressed that not only experts had flagged up but also various stakeholders and ordinary citizens. Fundamentally revising or abandoning the technology was not seriously considered; rather, the European Commission launched a Code of Conduct for research in the field after an extended public hearing.^11^ The way how nanotechnology had been implemented helped coining the term ‘Responsible Research and Innovation’ the European Commission subscribes to for other technological areas as well [30].^12^

When reference is made to nanotechnology in connection with SB, it is mostly regarding their common property of an emerging technology promising competitive advantages that, at the same time, should be introduced responsibly. For example, a range of high-ranking committees such as the US President's Committee on Ethics in Science and Technology [13] and the European Group on Ethics [46] have emphasised that addressing scientific uncertainties as well as concerns among the public early-on will be essential for the future of SB, even if there are currently few risks from a technical point of view.

The guiding image of the European Commission's interpretation of ‘Responsible Research and Innovation’ concerning ‘sensitive’ technologies (i.e. prone to elicit public unease) seems pervasive when it comes to emerging technologies. Taken the other way round, any technology that seemingly requires efforts at being introduced ‘responsibly’ may be considered potentially contested. With a view to the yet non-existing but anticipated protest, SB is obviously considered ‘sensitive’, and nanotechnology is seen as an appropriate example of how to successfully deal with a sensitive technology.

### 3.3 Information technology

Information or computer technology has held sway over the last decades in an unprecedented way. Hardly any technology (with the exception of steam or electric energy) had a similar impact on modern society ever. Computers govern virtually every aspect of modern life and caused an explosion in productivity. Initial resentments were overcome quickly, and IT has developed into a synonym for the most powerful, pervasive and, at the same time, ‘cool’ technology imaginable. Gadgets and toys galore have contributed to this image, and possessing the newest product has become the most relevant status symbol. There is a critical debate on aspects such as intellectual property, privacy or various effects of the Internet, to name but a few, but the technology as such is established beyond any question.

SB can be considered an information technology, too, only using a different medium, namely DNA base sequences rather than electrons. This said, SB is a varied field that hardly can be put under a single umbrella [47,2]. Nevertheless, protagonists stress the IT analogy, and many pertinent examples and apparent similarities between SB and IT appear in the literature. The analogies mostly refer to elements of the technologies themselves even if they derive from entirely different disciplines, as if their very nature were closely related – rather than the communities involved.

The closest link is established through the scientists involved – many if not most of the original protagonists in SB come from the IT sector.^13^ As part of their professional world-view, they frequently allude to IT construction elements such as integrated circuits, devices and systems, etc., when talking about biological entities such as genes, cells and organisms. In addition, they decidedly set out to apply engineering principles in biology, and they see this at the very heart of SB [48–51].

Consequently, and similar to mechanical, electrical or electronic engineering, technical standards are considered essential. Accordingly, they are necessary to realise the far-reaching claims of an entirely new way of doing biotechnology via constructing living organisms from black-boxed genetic building blocks.^14^ In the same vein, analogies to principles such as abstraction hierarchies or ways of information processing are abundant (e.g. [52]). Although not representative for the whole SB community, this approach has gained most prominence among researchers and successfully communicates its achievements to the media.

Another level of analogy is that of societal impact. Here, quantitative parameters for progress (‘Moore's law’) are applied in a similar way as in computer technology – speed and cost of technical performance develop exponentially in opposite directions [53]. Stages in the development of the technologies, their applications and their importance for economic competitiveness and social wellbeing are compared – SB is depicted as a potentially pervasive technology in its infancy, in a position similar to that of IT in the Seventies or Eighties, implying enormous prospects for applications to come. Information technology thus provides ample opportunities to exemplify the huge potential of the ‘next technological wave’ SB is said to initiate.

Apart from such bold statements, there are a number of indicative practices that link SB tightly to the engineering culture of applied computer sciences. One of the most remarkable is iGEM, a yearly students’ competition that is decisively egalitarian, does not require a lot of money to participate and is prone to elicit the enthusiasm of students.^15^ This competition has developed into a major public relation agent, spreading the message of the ‘coolness’ of doing SB among the young, not unlike competitions in computer engineering such as the Robocop. Finally, do-it-yourself communities (including ‘garage shops’) have been emulated from the field of IT in a demonstrative way. Arts’ exhibitions, film festivals and similar events contribute to solidify a not uncritical but still ‘cool’ image of SB not only among biotechnology students.

Another similarity is the emphasis on particular forms of intellectual property management applying open source regimes rather than patenting [54]. Similar to the open programming community, patents are abhorred and free sharing of ‘constructions’ advocated [55]. Remarkably, the IT link has been less dominant in the media reporting in Europe. Media analyses [39] revealed general engineering metaphors galore but few explicit IT references.

## 4 Comparators and their frames

The frames applied in describing and debating SB in analogy to bio-, nano- or information technology, respectively, are clearly different. The implications of SB being depicted along those lines differ as well. In fact, there are three entirely or, at least, partly different debates subject to which analogy is prevalent.

### 4.1 Biotechnology: technology as conflict^16^

For a long time, the debate on green biotechnology, was oriented at potential risks from the intentional or unintentional release of genetically modified organisms, its public perception and the political consequences thereof. This was the case both in the closed circles of lay consensus conferences (for example [56]) or ‘out there’ in the public sphere. The discrepancy between the US and Europe regarding the preferred terms of reference for risk from SB mirrors somehow the long-standing quarrel over the alleged risk from GM crops. They were put against expected productivity benefits in terms of competitiveness, and later also in terms of environmental gains and the fight against world hunger.

Over time, the frame in the debate of GM crops changed. Over the last decade, the range of dominant frames widened, in other words, it has become officially accepted that the debate is not solely on risk vs. economic benefit but on other issues as well. The most impressing change has taken place with the European Commission's decision to accept the discrimination between GM and non-GM crops on the basis of ‘freedom of choice’, which does not have anything to do with risk but with principles of the free market [57, p. 189 ff]. On the contrary, any reference to risk is explicitly rejected as risks are officially non-existent as long as the competent authorities in the member states and the relevant EU institutions have not substantiated them. Nevertheless, the overarching frame is still risk-related, which becomes obvious in the campaigning of environmental NGOs. While equity issues on a worldwide basis such as the control over natural resources get increasingly addressed, the ultimate argument is the risks of GMOs or, ultimately, uncertainty and the absence of a proof of non-risk. Similarly, in international trade negotiations, the position of the EU is argued in a similar risk-related way. One may therefore conclude that risk continues to be the dominant frame in the debate on green biotechnology, but on the margins it has been frayed to allow for other, especially economic frames to take over.

From time to time, an ethics frame appears in a special issue at stake such as equity. Interestingly though, ethics does not seem to play a dominant role in the biotechnology analogy of SB as references to one of the strongest possible ethical challenges, the ‘playing God’ metaphor, seem to be less attractive to the press and to members of the public than expert discourse sometimes suggests ([39,58]).

Experts and policy makers frequently address the possibility of a risk discourse on SB. A major implication from the expert orientation of such a discourse would be expert disagreements and allegations by a suspicious public of experts holding partisan interests. The biotech comparator is also a toehold for environmental NGOs to step in and criticise SB as an even more ambitious attempt at industry gaining control over natural resources at the expense of citizens and communities being afflicted with risk, combining risk and ethics frames in a peculiar but well-tried perspective of David versus Goliath [36,59]. An extension of old biotech debates is to be expected if SB is going to be framed familiarly as a risk issue.

If one would try to capture in a single term what technology is displayed like when the comparator of (green) biotechnology is applied then the word ‘conflict’ comes to mind. Thus, an emerging technology such as SB would be conceptualised primarily as potential source of conflict. This notion of ‘technology as conflict’ shapes expectations and imagined futures and prompts important actors to act accordingly: to emphasise potentially conflicting issues or to seek ways how to avoid, in a pro-active way, conflict to materialise. This has also repercussions for the general conceptualisation of the role of the public as a factor to be taken very seriously.

### 4.2 Nanotechnology: technology as progress

In the analogy to nanotechnology, an economic frame of progress dominates largely over a risk frame. The latter is acknowledged as being relevant but currently dealt with mostly on a scientific level, emphasising uncertainty over potential risks and stressing the need for more research.^17^ The composition of frames is similar to that encountered in the debate on green biotechnology, but in nanotechnology the emphasis is more on economic benefits and hence, the technology appears in a much brighter light.^18^ To be caught in a single term, technology in the sense of nanotechnology is equivalent to a promise for progress that needs to be protected and turned into reality.

The bright image had not always been dominant. There are numerous accounts of politicians and scientists saying that nanotechnology might be met with the same suspicion as biotechnology, and that this must be prevented. Implementing the technology in a ‘responsible’ way eventually developed into the overall rationale. The aim was, to speak in terms of frames, to put the risk frame at bay and replace it with an economic frame, emphasising the prospects. This went along with an emphasis on ‘upstream engagement’ in the double meaning already referred to: on the one hand, engaging the public with the aim to spur enthusiasm about the technology and to increase the trust in those in charge of steering it. On the other hand, to provide the means that members of the public can engage and take an active part in influencing the development of the technology. The conceptualisation of the public therefore remains ambiguous. This double meaning of upstream engagement creates a tension between theoretical aspirations and practical constraints of activities, which at times reproduce the old ‘public understanding of science’ patterns of expert orientation and lay exclusion [62].

Similarly, Groves [63] discerned two different rationales for stimulating early public engagement in nanotechnology: restoring trust and building robustness. Both pursue the aim of reducing uncertainty at an early stage of technology development that might hinder innovation. Accordingly, the former aims at preventing public distrust in key actors and technological processes without affecting technology development significantly. The latter seeks to shape technology in a socially robust way, irrespective of what people think of those in charge. Framed by assumptions about the nature of innovation deeply embedded in the elite's perceptions, Grove identified the former to have lain behind many UK Government-sponsored experiments with upstream engagement, effectively preventing the latter from becoming reality despite opposite rhetoric. Also with regard to SB, both understandings seem to co-exist among decision-makers rather silently, giving rise to misunderstandings.

It remains speculative whether the seeming lack of open conflict over nanotechnology ‘out there’ is due to the efforts at upstream public engagement. It is equally unclear whether these activities had any influence on the actual trajectory of nanotechnology development whatsoever. In analogy, it also remains unclear whether upstream engagement will have any effect on a potential future debate on SB as it might also be stricken with a double understanding of ‘engagement’. Whatever the frame will be, will it be modulated by a quest for sustainable or socially robust shaping of the technology? Or will it follow an extended, perhaps reflectively refurbished ‘public understanding of science’ approach having moved upstream?

### 4.3 Information technology: technology as gadget

With information technology (IT) as a comparator, the ‘coolness’ factor and the notion of personal benefit are paramount. Risk is an issue only with respect to intentional misuse, like in cybersecurity. Overall, a seemingly egalitarian attitude clearly prevails, along with an imagination of universal feasibility – everything will be possible. Put in a single term, technology appears as the means providing the latest gadget, the most advanced tool, the coolest toy, together with the promise of technological and social freedom. The public appears as potential consumers, on the one hand, and as enthusiast participants in the play that need to be taken on board.

The do-it-yourself (DIY-Bio) community, although partly recruited from SB scientists fed-up with institutional constraints (See the paper by Delgado in this issue) might be considered part of the system, as it supports the IT analogy on yet another level. The coolness factor is supported by frequent allusions to metaphors of engineering and playing in the press, although an explicit link to IT is rarely provided. One may speculate that the analogy is not very salient to members of the (European) public, at least not in the way SB protagonists from the IT sector propagate it. If it would become more prominent, it might push back the analogy to biotechnology and foster new frames in a debate to come. Possibly, such a debate would emulate the preoccupation with data processing technologies abundant today, and hence popularise a new aspect that had been largely absent in public debates ‘out there’ about various forms of biotechnology so far. This may coincide with the advent of systems biology as a new discipline within the life sciences, which also builds heavily on bioinformatics, and promote the colonisation of biology by computer sciences.

If SB is going to be debated in a similar way as IT, we may expect a strong emphasis on empowerment. Such an emphasis might be observed already with respect to intellectual property management, where many stakeholders prefer open source solutions to patenting [54,55]. This may be interpreted as an anti-establishment position, but it may also reflect pragmatic aspects of everyday research conditions. The biological ‘open space’ analogy to the internet remains elusive, though. The internet had a profound impact on the image and the range of personal applications of IT; it seems to be unique, so analogy building might be restricted to the mere technical engineering aspects.

Taken together, different possible futures are linked to the three comparators. The visions attached are unrolled along the lines of recent and on-going developments in the comparator technologies. From an upstream position it is impossible to assign more or less plausibility to any of the comparators – they all have certain persuasiveness. It remains a matter of competition which of them (or maybe another one) may turn out to be dominant and determine the frames of future debates both in engagement activities and in the public sphere.

## 5 Stimulating a debate on synthetic biology – what, and what for?

Despite many initiatives, ‘stimulating an open debate’ might be a difficult task. Issues of a technology like SB that cannot properly be linked to the everyday world of citizens mostly remain a matter for a few interested, so specially set-up engagement events prevail in such a case. However, the comparison with other technologies facilitates the imagination of pertaining futures, raises the expectation for particular benefits and provides a range of relevant problem settings, as if synthetic biology was ‘like’ green biotechnology, nanotechnology or information technology. These comparators suggest dominant frames, i.e. relevant issues and arguments. They entail normative decisions to be made, but they do not pre-determine them. Choosing a particular comparator does not necessarily impinge on the conclusion, in an engagement exercise, whether the technology should be supported or prevented from happening. Rather, the reason given why SB should be supported or opposed might differ.

If a comparator becomes dominant, i.e. obvious to many experts, stakeholders and members of the public it might influence the course of a debate ‘out there’ through suggesting one or more dominant frames. They will reflect the encompassing nature of the debate through their implicit conceptualisation of the public: ‘technology as conflict’ goes along with the public to be taken seriously; seen through the glasses of ‘technology as progress’, the public appears as an entity to be mastered through appropriate means; and with ‘technology as gadget’ the public is seen as a player in the technology's own team, so to say.

The most potent trigger of a debate in the public sphere many individuals would engage in is a mobilising controversy. So far, such controversies mostly emerged over risk issues, and the comparator with the longest history under a risk frame is agricultural biotechnology. Thus, with this comparator we may expect a debate like over GM crops. This, however, would be what upstream attempts at stimulating a public debate are thought to prevent. This leads to the conclusion that being successful in stimulating a public debate means failure in preventing controversy. In contrast, benefit as the dominant frame has far less potential to elicit a controversy. With nanotechnology as the comparator, a controversial debate becomes unlikely, because risk issues would then be contained and delegated to expert circles. With information technology, a risk debate would be even less likely, although particular issues (such as privacy aspects) have raised controversies out of an application perspective, but at a much later stage in technology development.

For SB this means avoiding the biotechnology comparator, and getting away from the risk frame, might appear desirable for those promoting the technology, but less desirable for those who aim at stimulating a public debate. This has been a recurrent problem in experimental forms of public debate such as participatory procedures: participants are difficult to find and to engage, they often discuss half-heartedly in an artificial setting, and the result is more than meagre with respect to impact. Overall, the legitimisation value of such procedures is low [22]. The example of nanotechnology is telling: initiatives at upstream engagement seem to have succeeded in eradicating any traces of a potential risk debate in a seemingly over-addressed public. Again, this may be seen as a success regarding the public image of the technology, but not in terms of stimulating a public debate.

This leaves similar initiatives with SB appear in an ambivalent light. When promoting societal deliberation over new technologies, some advocates may have an instrumental approach in mind, namely to evade problems for innovation from an ignorant public through inducing a well-controlled debate. Others may wish to promote democracy, by enabling citizens to have a voice. Still others may wish to reap gains in rationality for a decision over the trajectory of a technology. There are arguments for all agendas, but they partly oppose each other. Therefore, when stimulating a public debate, the conceptualisation of the public should be unambiguously clear, and the respective agenda should be laid open even if this would result in a conflict. Such conflict, after all, may be the best stimulus for a debate.

## Footnotes

1 We read this as indicating the inability to anticipate the properties of future products and processes as well as the nature and distribution of benefits and risks, rather than as an indication that the author(s) are unaware of the difference between uncertainty and risk, their possible understandings and their respective relevance to different parties.

2 Gaskell et al. [21, p. 120] described the ‘public sphere’ as “a triangle of mediation between policy, the media and public perceptions.”

3 In the following, we will apply the term ‘debate’ also for deliberation and dialogue, being aware that there are differences indeed. However, we think the term ‘debate’ best captures the more engaged way of discussing an issue.

4 The basic frames Dahinden [28, p. 210 ff] identified are conflict (equivalent to risk), profitability and progress (two aspects of the economic frame), moral (which Bogner [17] called ethics), and personalisation, i.e. personal affectedness, which is not a discourse rule, however.

5 An example was an early stakeholder dialogue on transgenic plants at the Wissenschaftszentrum Berlin [29], where the organising scientists deliberately set a risk frame, which the participating NGOs initially accepted but later expanded to include a combination of economic and ethical frames. Consequently, they left the table because they no longer could maintain their position under the dominant risk frame. This also shows that a technology may be opposed under an economic frame.

6 A telling metaphor from molecular biology could be the prion protein acting as a chaperone: guiding a nascent polypeptide chain in acquiring the three-dimensional structure of the prion.

7 Choosing the issue and the argument to dismiss it already defines a frame. For example, researchers defended stem cell research early-on using ethical arguments [24].

8 Even in the US, the issue of GM food and its labelling seems to enter the agenda: http://www.guardian.co.uk/commentisfree/2012/jun/13/california-gm-referendum-change-america-food.

9 See e.g. the ‘nanotrust’ dossiers by ITA, http://nanotrust.ac.at/dossiers.html (retrieved 21.01.12).

10 For example, Rita Colwell, then director of the US National Science Foundation, said in an interview about nanotechnology: “… we can’t risk making the same mistakes that were made with the introduction of biotechnology.” (Anna Fazakerley, “Nanotechnology won’t suffer GM fate”, Times Higher Education Supplement, 27 June 2003, http://www.timeshighereducation.co.uk/story.asp?sectioncode=26&storycode=177714 retrieved 21.01.12).

11 Issues and Options on the Path Forward With the European Commission Code of Conduct on Responsible N&N Research, www.nanocode.eu/files/NanoCode-MasterPlan.pdf.

12 Ironically, the extent of activities sometimes seems to outperform the willingness of the public to participate or being educated – participatory events often suffer from difficulties in recruiting. In addition, their experimental setting provides an atmosphere of detachment from reality that put participants in the role of guinea pigs rather than a jury [22]. A reason may be that participation is not intended to exceed dialogue towards influencing the technology trajectory; hence, the exercises necessarily remain somewhat sterile.

13 For example, Randy Rettberg, the founder of the iGEM competition, is one of the fathers of the Internet.

14 See the BioBricks homepage http://BioBricks.org/programs/technical-standards-framework/.

15 http://igem.org/Main_Page (retrieved 21.01.12).

16 The denomination ‘technology as …’ does of course not imply that conflict is confined to biotechnology, progress to nanotechnology or playfulness to information technology. It is just an attempt at catching the main ‘taste’ of the frame in a single term.

17 The recent proposal to adopt a ‘CEA Assessment’ to determine the risk of synthetic biology [60] sheds a light on how experiences with and instruments applied in dealing with nanotechnology are emulated in SB even without mentioning their origin.

18 There is an allusion to an ethics frame as well: the debate on human enhancement introduced by the proponents of ‘converging technologies’ [61] frequently referred to nanotechnology. This debate, however, for a long time had little repercussion in Europe, and in the US it was more or less confined to a community of non-mainstream ‘transhumanists’. As a comparator, it cannot be said to have played a decisive role.
